# Endovascular Treatment of Intracerebral Giant Cell Arteritis

**DOI:** 10.3389/fneur.2020.00287

**Published:** 2020-04-16

**Authors:** Claus Z. Simonsen, Lasse Speiser, Ib Tønder Hansen, David Jayne, Paul von Weitzel-Mudersbach

**Affiliations:** ^1^Department of Neurology, Aarhus University Hospital, Aarhus, Denmark; ^2^Department of Neuroradiology, Aarhus University Hospital, Aarhus, Denmark; ^3^Department of Rheumatology, Aarhus University Hospital, Aarhus, Denmark; ^4^Department of Medicine, University of Cambridge School of Clinical Medicine, Cambridge, United Kingdom

**Keywords:** giant cell arteritis, stroke, endovascular therapy, immunosuppression, vasculitis

## Abstract

**Background:** Giant cell arteritis (GCA) is the most common primary systemic vasculitis predominantly affecting large and medium sized vessels. In rare cases, the vasculitis can affect the vessels of the brain.

**Results:** We describe four cases of GCA with involvement of the cerebral vessels causing stroke. These cases were unresponsive to aggressive immunosuppression and we opted to treat with endovascular balloon dilatation of the stenotic areas. The procedure was safe. The four patients were treated in nine sessions and a total of 16 vessels were treated. We observed two dissections with no clinical influence on the patients.

**Discussion:** In patients with stroke due to progressive GCA that is non-responsive to immunosuppression, endovascular therapy is feasible.

## Background

Giant cell arteritis (GCA) is the most common primary systemic vasculitis. It predominantly affects large and mediums sized arteries, typically the aortic arch and its branches, and branches of the external carotid artery. Women are affected 2–3 times more often than men. The classical clinical presentation includes headache, scalp tenderness, jaw claudication, visual disturbances, fever, and weight loss. Laboratory results show increased inflammatory markers. Biopsy of the superficial temporal artery can show inflammation of the vessel wall (1). Involvement of the intracerebral arteries and stroke have been described but are very rare. Treatment of GCA is medical. Endovascular treatment has only been attempted very rarely (2).

## Patients and Methods

We reviewed our cases of GCA with stroke in the period of 2015–2018. The diagnosis of GCA was clinical and followed the definition from American College of Rheumatology (3) demanding three of five criteria: (1) Age >50 years. (2) New headache. (3) Temporal artery tenderness. (4) Erythrocyte sedimentation rate >40 mm/h. (5) Positive biopsy of the superficial temporal artery.

We identified seven patients. Our approach to treatment was first to give high dose glucocorticoid and 2/7 had remission of symptoms on prednisolone alone. In a third case, we added pulses of i.v. cyclophosphamide (CPM) which stopped progression.

In the remaining four cases, where the disease was progressing with new transient ischemic attacks (TIA) or strokes, we chose to proceed with balloon angioplasty and CPM to suppress inflammation.

**Figure 1:**
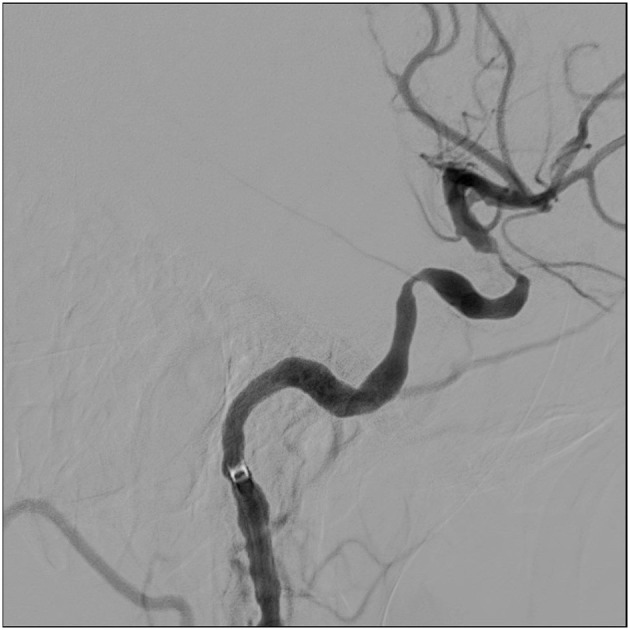
Digital subtraction angiography of case 1, lateral view of the left internal carotid artery. Two stenosis are seen.

**Figure 2:**
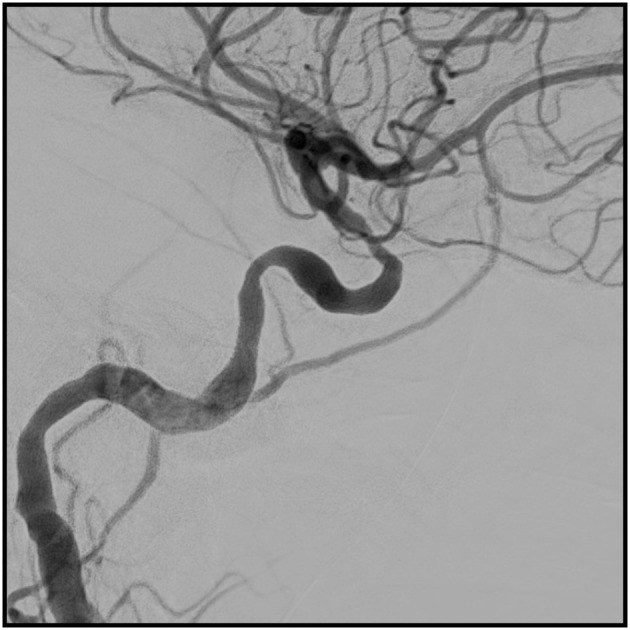
The same artery after balloon dilatation.

Balloon angioplasty was performed in three patients (case 1, 2, and 3, see below) with a non-compliant balloon catheter (Gateway, Stryker, Kalamazoo, MI). The stenoses were dilated to a maximum of 80% of the estimated normal vessel diameter, with at least two repetitive dilatations with successively increasing atmospheric pressures (see Figures 1, 2). The immediate angiographic results were excellent in all cases with an increase of the vessel diameter to 70–80% at the level of the stenosis.

In two of the cases (case 3 in the first treatment round and in case 4) it was not possible to cross the stenotic lesions by the balloon catheter. Angioplasty was instead performed with an adjustable remodeling mesh device, the Comaneci stent (Rapid Medical, Yokneam, Israel), in three stenoses at the level of the siphon and one V4 stenosis. We saw good results in the three siphon stenoses (a triple increase of the diameter in two cases and a double increase in one) and a modest effect on the V4-stenosis with an increase of the diameter of 20%.

Angioplasty was repeated twice in two patients and three times in one patient, with intervals of 2 weeks and 3 months. Examples of angioplasty from all the cases can be seen in Supplementary Materials.

## Cases

### Case 1

A 65-year old woman with no vascular risk factors was admitted with sudden aphasia and decreased sensation of the right arm. The patient complained of two episodes during the previous week with weakness of the right arm and 2 days later weakness of the left arm. Both episodes lasted 20 min. Also, she had experienced headache, jaw claudication and fatigue for the last 2 months.

Diffusion-weighted MRI showed bilateral ischemic lesions and MR angiography showed stenosis of both internal carotid arteries (ICAs) at the level of the carotid siphon. Cerebrospinal fluid (CSF) was normal. Oral high dose prednisone (1 mg/kg) was started.

The headache and jaw claudication resolved on this treatment, but the patient had progressive episodes of aphasia. Glucocorticoid treatment was increased to methylprednisone 1 g i.v. daily for 3 days followed by oral prednisone 100 mg daily with a taper. Tocilizumab was added, but the patient continued to suffer TIAs and small strokes resulting in worsening aphasia.

Ten days after admission, balloon dilatation was performed on the left ICA. Figures 1, 2 show a digital subtraction angiography of the stenosis before and after dilatation. She was followed with transcranial color-coded sonography (TCCS). Repeat balloon angioplasty of the left ICA was performed 1 month later when TCCS showed worsening of the stenosis.

CPM was started (first dose i.v. 15 mg/kg and then 2 mg/kg orally daily). This was given for a total of 6 months and the patient was transitioned to methotrexate. The patient had no further strokes. The 90-day outcome for all the patients is found in Table 1.

**Table 1:** Summary of the key findings and treatment details in the patients.

**Patient #**	**Initial ESR**	**Biopsy of STA**	**PET scan**	**Number of treatments, number of vessels treated and complication**	**Medical treatment besides prednisone**	**Time between first stroke and treatment**	**90 day mRS**
1	46	Positive	Positive. FDG uptake in maxillary and vertebral artery	2 treatments, 1 vessel (left ICAx2). No complications	I.v. CPM 15 mg/kg x1, then oral CPM 2 mg/kg daily for 6 months	10 days	2
2	57	Positive	None	3 treatments, left ICAx3, right ICAx2. 1 dissection	I.v. CPM 15 mg/kg x6	2 days	3
3	22	Positive	Positive. FDG uptake in maxillary, vertebral and STA	2 treatments. Left ICAx2, right ICAx1, left vertebral x1. No complications.	I.v. CPM 10 mg/kg x6	2 weeks (progressive apraxia and apathy)	1
4	54	Non-specific	None	2 treatments. Left ICAx2, right ICAx2, left vertebral x1, 1 dissection	I.v. CPM 15 mg/kg x6	Acute treatment (TIA 14 days prior.)	4

*CPM, Cyclophosphamide; ESR, Erythrocyte Sedimentation Rate; FDG, Fluor-deoxy-glucose; ICA, Internal carotid artery; mRS, modified rankin scale; PET, Positron emission tomography; STA, Superficial temporal artery; TIA, Transient Ischemic Attack*.

### Case 2

Sixty-four-year old woman with a past medical history of hypertension and diabetes. She presented with severe headache 3 months before she was diagnosed with GCA. Two days after starting high dose oral glucocorticoids, she was admitted with wordfinding difficulties. Angiography showed bilateral stenosis of the intracerebral ICAs. Glucocorticoid treatment was increased to methylprednisone 1 g i.v. daily for 3 days followed by oral prednisone 100 mg daily with a taper. She was treated with balloon angioplasty of the left ICA and had a small dissection. She was started on i.v. CPM (15 mg/kg, every 3 weeks.)

She was admitted again 2 weeks later with apathy and gait disturbances. A further MRI scan showed progressive bilateral anterior infarcts and she was again treated with balloon angioplasty of both intracerebral ICAs. She was readmitted 1 month later after a fall. No new lesions were seen on MRI, but both ICAs were dilated again as a precaution. She had no new documented strokes after the second treatment.

Her course was complicated with pneumocystis pneumonia successfully treated with Sulfamethoxazole/Trimethoprim. After 6 cycles of i.v. CPM, she was transitioned to methotrexate.

### Case 3

**Figure 3:**
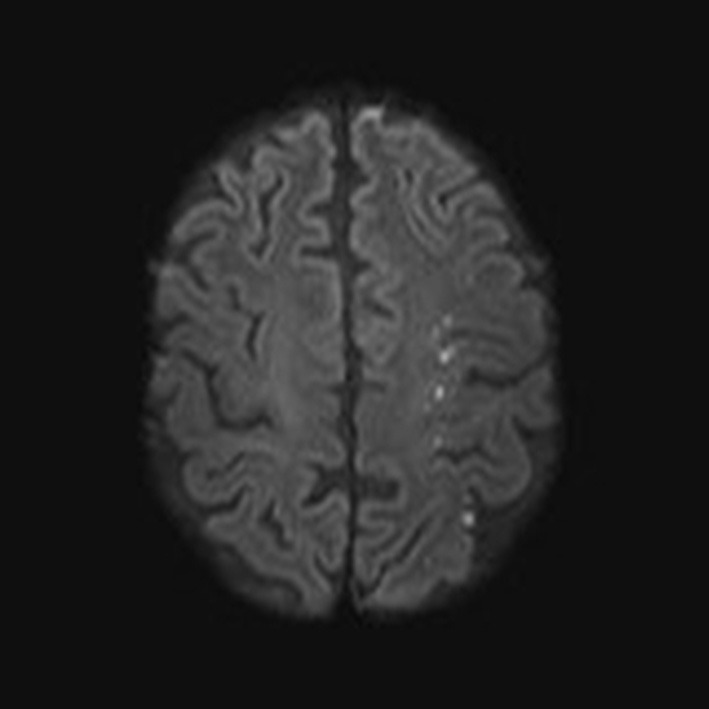
Ischemic lesion in the left hemisphere distributed in the watershed area in case number 3. This indicates an embolic source or compromised flow in the left internal carotid artery.

**Figure 4:**
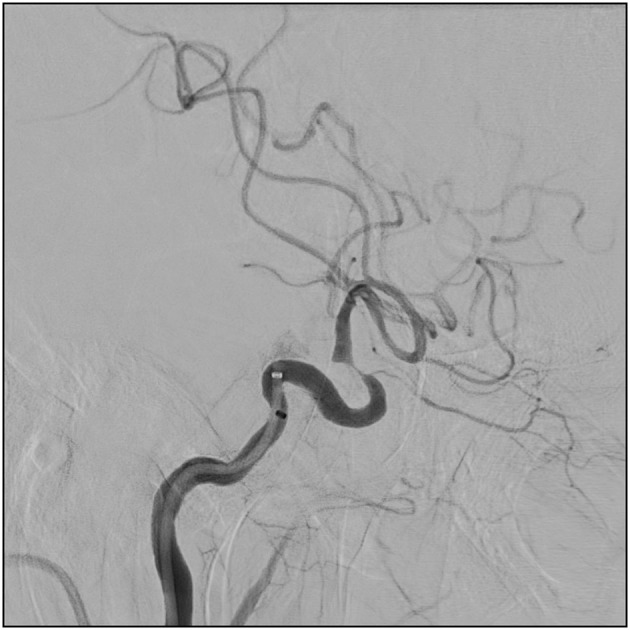
This is the stenotic lesion in the internal carotid artery in case number 3.

Seventy-two-year old male with a history of hypertension, diabetes, smoking, and colon cancer. He was admitted with sudden vision loss on the right eye, increasing tiredness, headache, weight loss of 7 kg, and isolation from social activities. A diagnosis of GCA was made and the patient was started on methylprednisone 1 g i.v. daily for 3 days followed by oral prednisone 100 mg daily with a taper. When he reached 20 mg daily, he was readmitted due to forgetfulness, speech difficulties, and apraxia. CSF was normal. An MRI revealed ischemic lesions in the watershed areas in the left hemisphere (Figure 3). He was re-started on high dose glucocorticoid (1 g methylprednisone i.v. daily for 3 days followed by oral prednisone 100 mg daily with a taper) and was treated with balloon angioplasty of a stenosis in the left carotid siphon. This treatment was 110 days after the initial presentation. Figure 4 illustrates the characteristic stenotic lesion.

Two weeks after the initial angioplasty he was readmitted, again with a change in personality and apathy. He was again treated by angioplasty of both intracranial ICAs. After the first endovascular treatment, he was started on CPM 10 mg/kg every 3 weeks for a total of 6 treatments and then transitioned to methotrexate. He was followed in the outpatient clinic with no new complains.

### Case 4

Seventy-one-year old woman with only mild hypertension. She was seen with a TIA (left sided weakness), but then admitted 2 weeks later with severe left sided weakness. She was taken for thrombectomy and the angiography revealed multiple stenoses. Balloon dilatation was performed on the right intracranial ICA. Retrospectively, she complained about headache and jaw claudication 1 month prior to the TIA. CSF was normal. She was started on 1 g of methylprednisone i.v. daily for 3 days followed by oral prednisone 100 mg daily with a taper. A few days after the initial treatment, paresis on the left side became worse and a new MRI revealed an infarct in the pons. She was taken for angiography again 7 days after the first treatment, and stenosis on both the carotids and the left vertebral arteries were treated. The dilation of the vertebral was complicated with a dissection.

She was started on i.v. CPM 15 mg/kg every 3 weeks for 6 treatments and transitioned to methotrexate. She had no new strokes after this.

## Discussion

In this case series, we present patients with strokes on the basis of GCA. Some of the cases progressed regardless of high dose glucocorticoids and intervention with balloon angioplasty of cerebral vessels was performed as a rescue therapy.

The intervention was done with minimal complications. A total of 16 vessel-treatments were performed and we only saw two dissections evident on angiography, which did not cause any symptoms. Our impression was that rescue balloon angioplasty is safe and maybe beneficial together with immunosuppression after steroid failure.

Stroke has been described before as a complication of GCA. In a new French study, the patients with GCA and stroke were more often older and more frequently men compared to patients with GCA and no strokes (4). This is in contrast to the sex distribution in GCA, where women are affected more often. Also, the frequency of ocular involvement was higher in the patients who subsequently had stroke. Another large retrospective study also found a high rate of ocular ischemia and a higher age among patients with stroke due to GCA, but a more even sex distribution (5).

Intracerebral stenosis in GCA can respond to high dose glucocorticoids (6). But it has been reported previously that the progression of strokes in some cases do not respond to glucocorticoid treatment (7). Progression of strokes has also been described after initiation of glucocorticoid treatment and a clinical response to the systemic symptoms (8, 9). After the success of endovascular therapy in acute ischemic stroke (10) and relatively few side effects, it was a logical step to try this option in patients with progressive symptoms and large vessel stenosis on inflammatory background.

We have only found balloon dilatation and stenting described before in three single cases (11–13). The use of stent retrievers in the treatment of cerebral vasospasm in patients with subarachnoid hemorrhage has been described recently (14) and the technique was adopted in cases due to failure of performing balloon angioplasty.

As aggressive medical treatment beyond high dose glucocorticoid, we chose CPM. This drug is generally chosen to control severe cases of cerebral vasculitis. The patient in case 1 was treated with the interleukin-6 receptor inhibitor tocilizumab. This drug has shown to be effective in obtaining remission in patients with GCA together with lower cumulated doses of glucocorticoid (15). In our case, the patient's strokes continued after initiation of tocilizumab.

The limitations to our study are that the patients were not prospectively collected and treated on a case by case nature.

Our aim with this paper is to report that progression of neurological symptoms in a patient with GCA should lead to aggressive treatment with high dose glucocorticoid and CPM. Endovascular therapy with angioplasty for severe symptomatic stenosis in this setting can be considered and appears to be reasonable safe.

## Data Availability Statement

All datasets generated for this study are included in the article/Supplementary Material.

## Ethics Statement

Written informed consent was obtained from the patients for the publication of this case report.

## Author Contributions

CS and DJ researched literature and conceived the study. CS wrote the first draft of the manuscript. All authors were involved in managing the patients, reviewed and edited the manuscript and approved the final version of the manuscript.

### Conflict of Interest

CS is supported by a research grant from Novo Nordisk Foundation. The remaining authors declare that the research was conducted in the absence of any commercial or financial relationships that could be construed as a potential conflict of interest.

## Supplementary Material

PRESENTATION 1 Here are examples of digital subtraction angiography of the 4 treated patients before and after balloon angioplasty.
