# Supplementary figures and images for: Endovascular Treatment of Intracerebral Giant Cell Arteritis
Source: Front Neurol. 2020 Apr 16;11:287. doi: 10.3389/fneur.2020.00287 (PMC7177021; doi:10.3389/fneur.2020.00287)

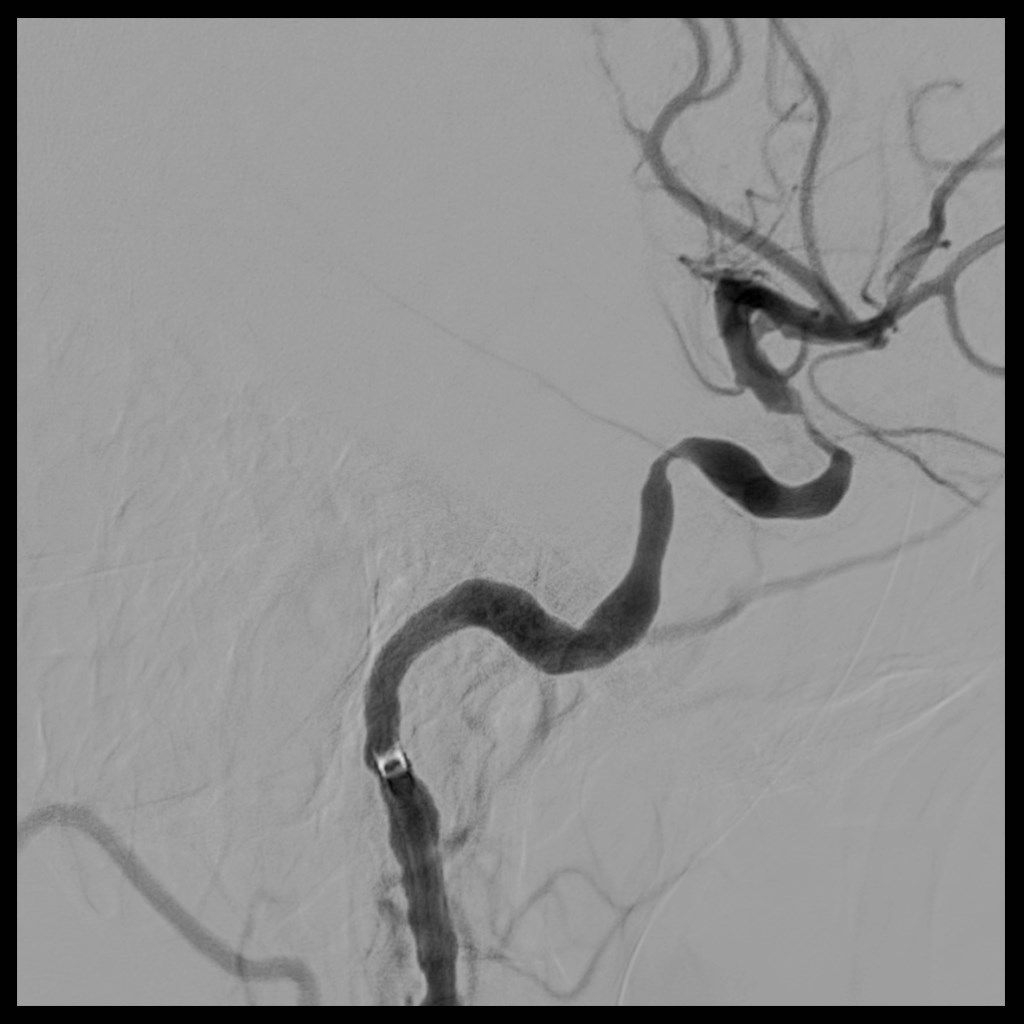

Supplement: PRESENTATION 1 [file Presentation_1.ZIP › 1a_Patient1_prePTA.jpg]

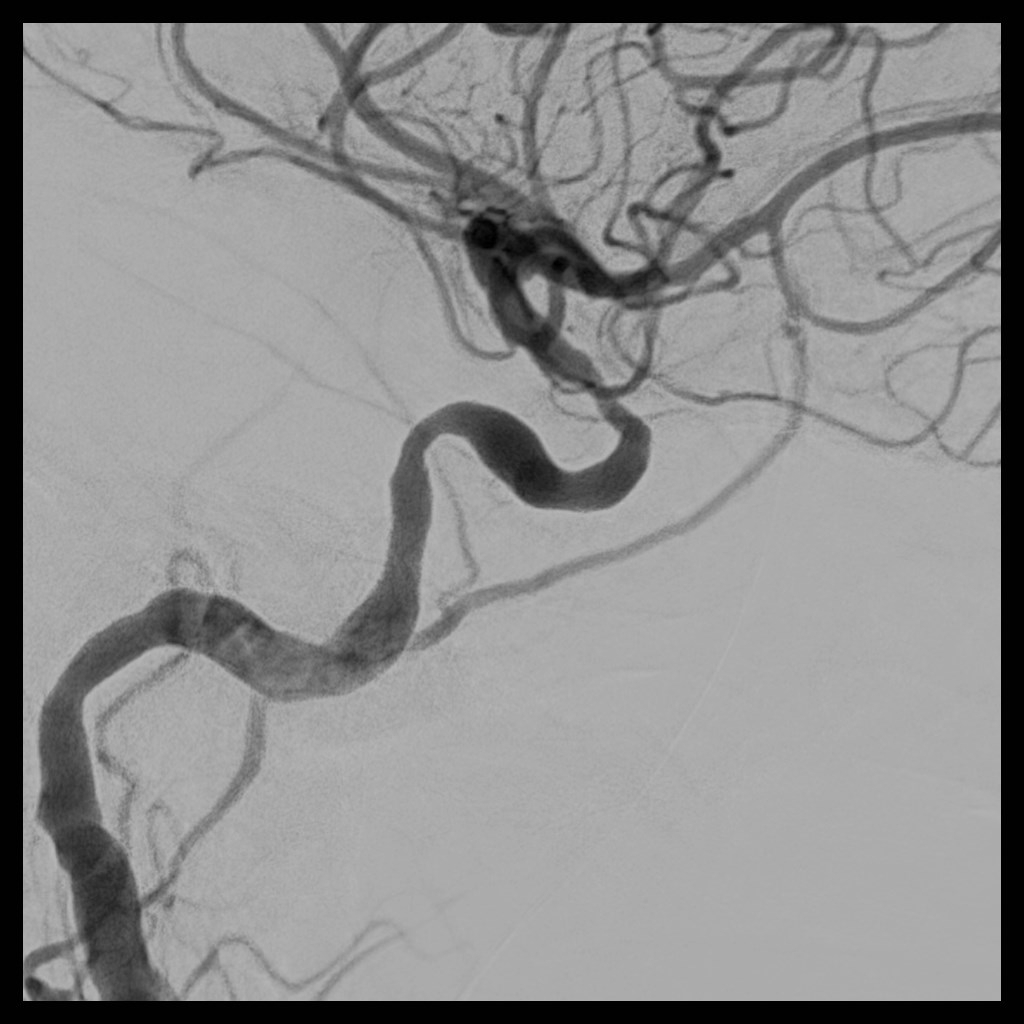

Supplement: PRESENTATION 1 [file Presentation_1.ZIP › 1b_Patient1_postPTA.jpg]

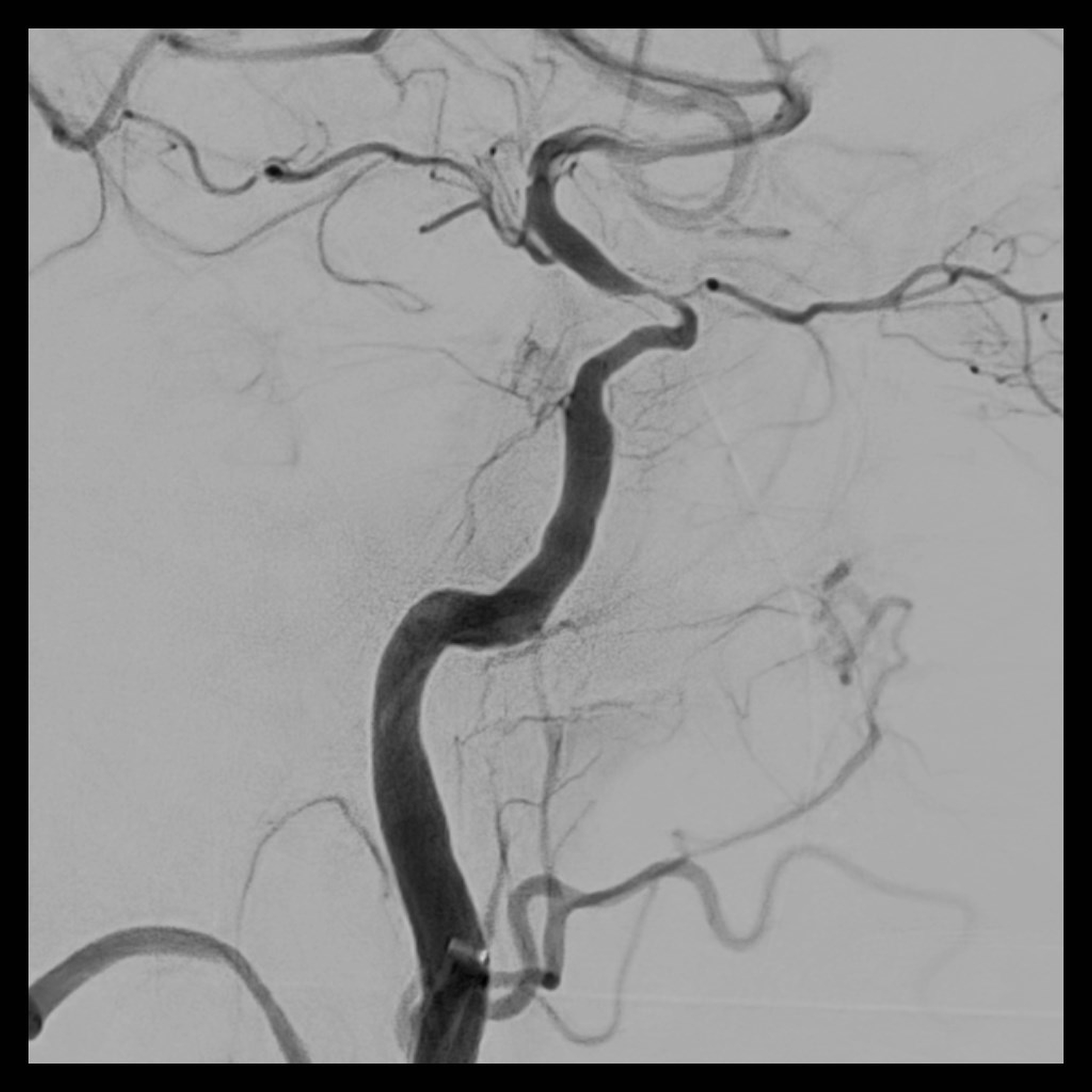

Supplement: PRESENTATION 1 [file Presentation_1.ZIP › 2a_Patient2_prePTA.jpg]

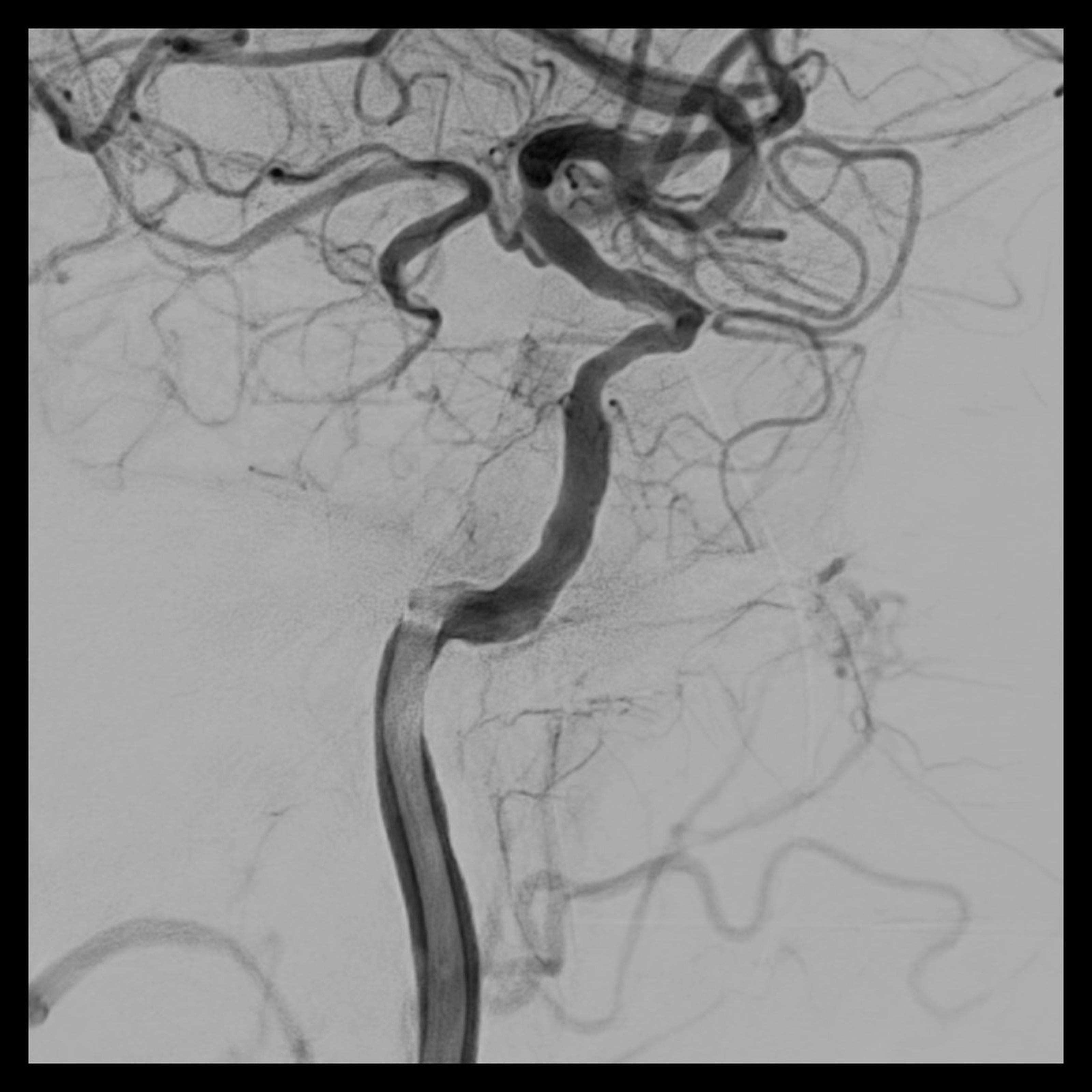

Supplement: PRESENTATION 1 [file Presentation_1.ZIP › 2b_Patient2_postPTA.jpg]

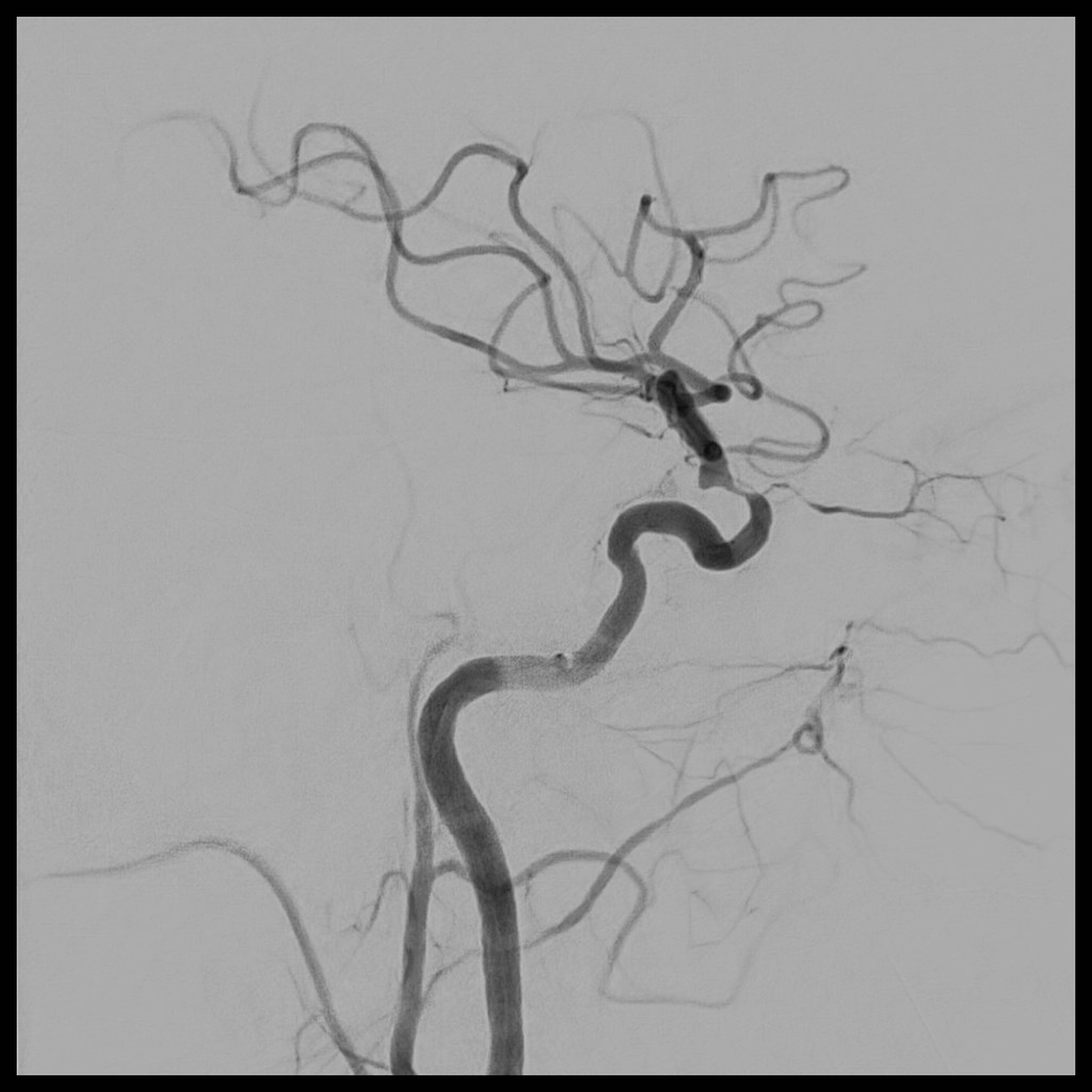

Supplement: PRESENTATION 1 [file Presentation_1.ZIP › 3a_Patient3_prePTA.jpg]

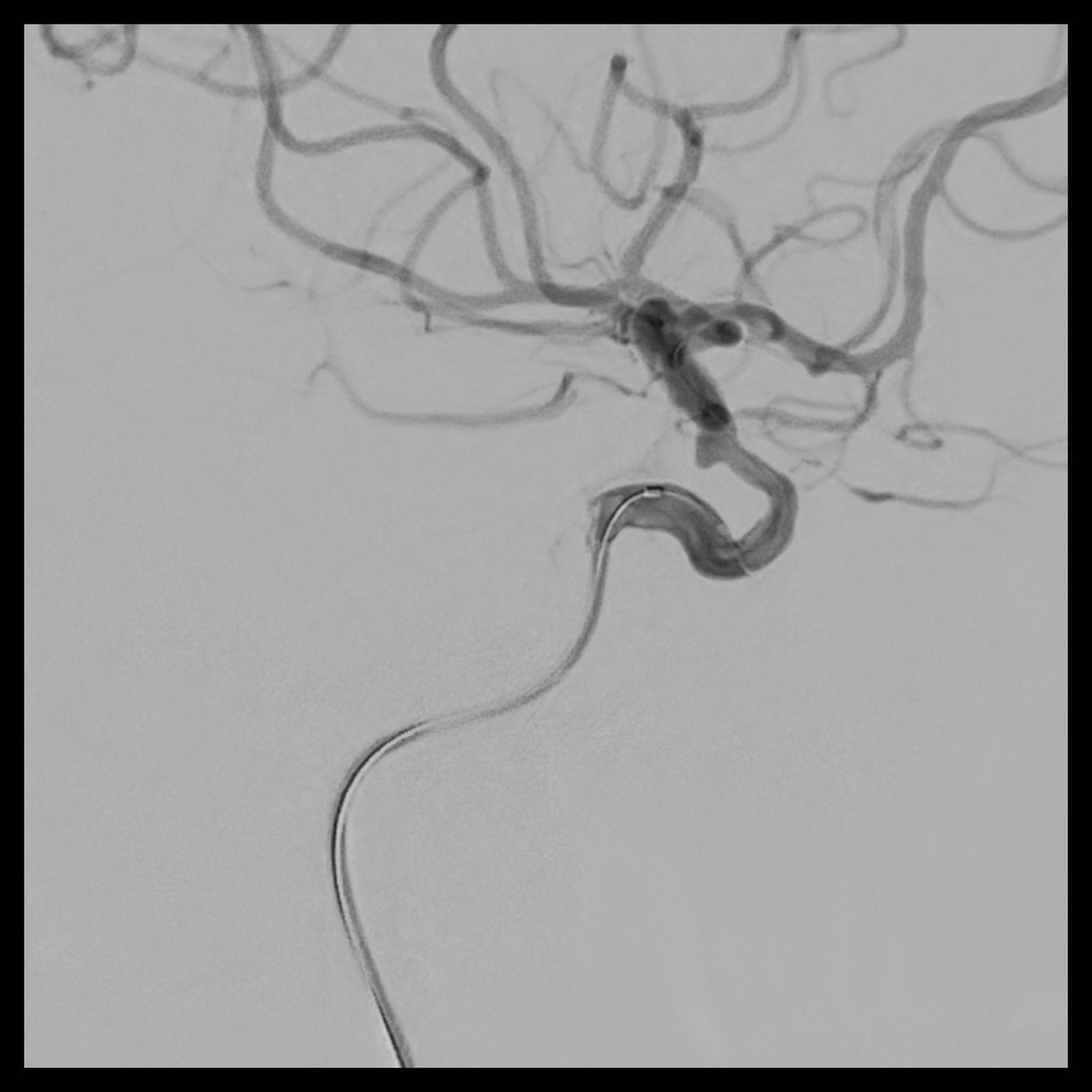

Supplement: PRESENTATION 1 [file Presentation_1.ZIP › 3b_Patient3_postPTA.jpg]

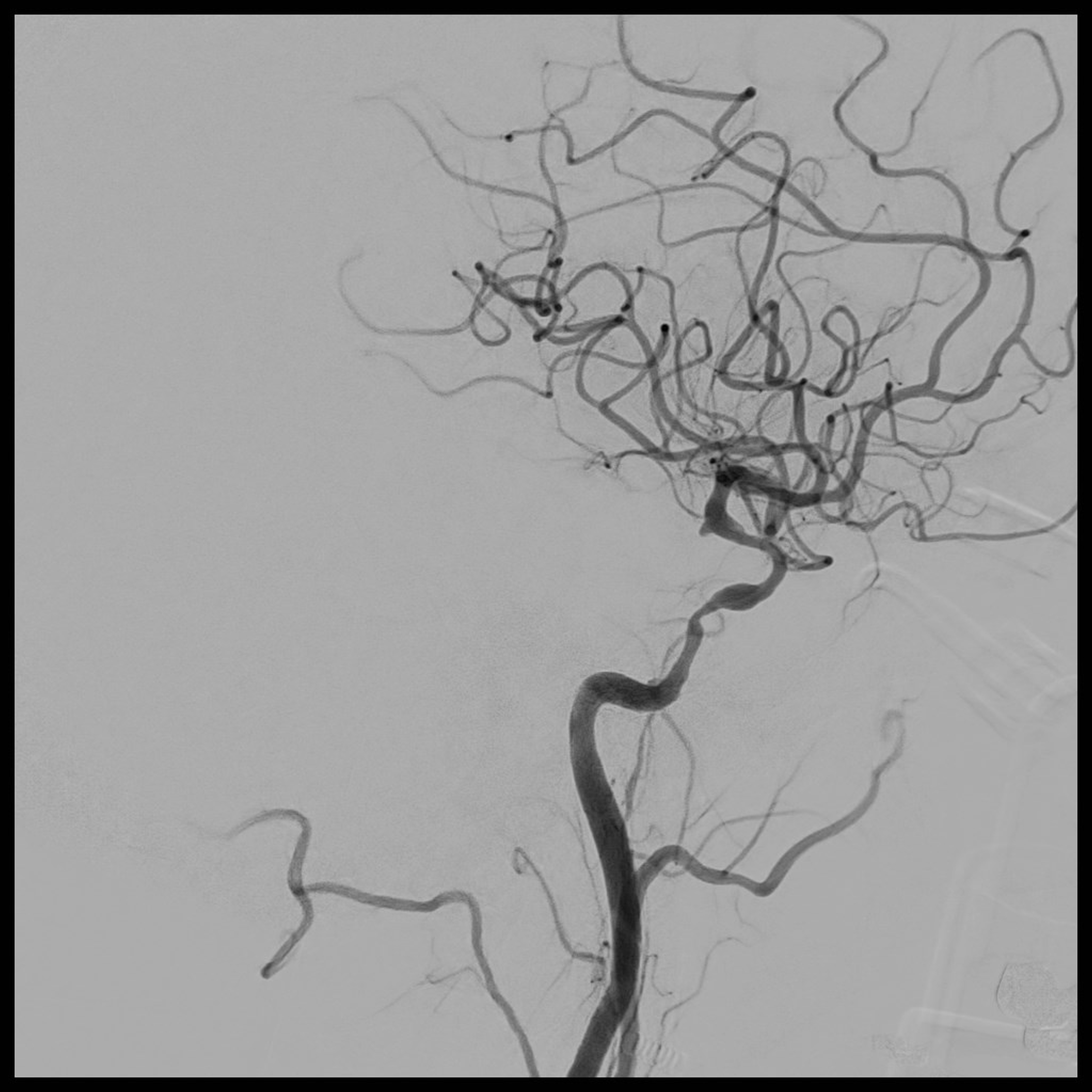

Supplement: PRESENTATION 1 [file Presentation_1.ZIP › 4a_Patient4_prePTA.jpg]

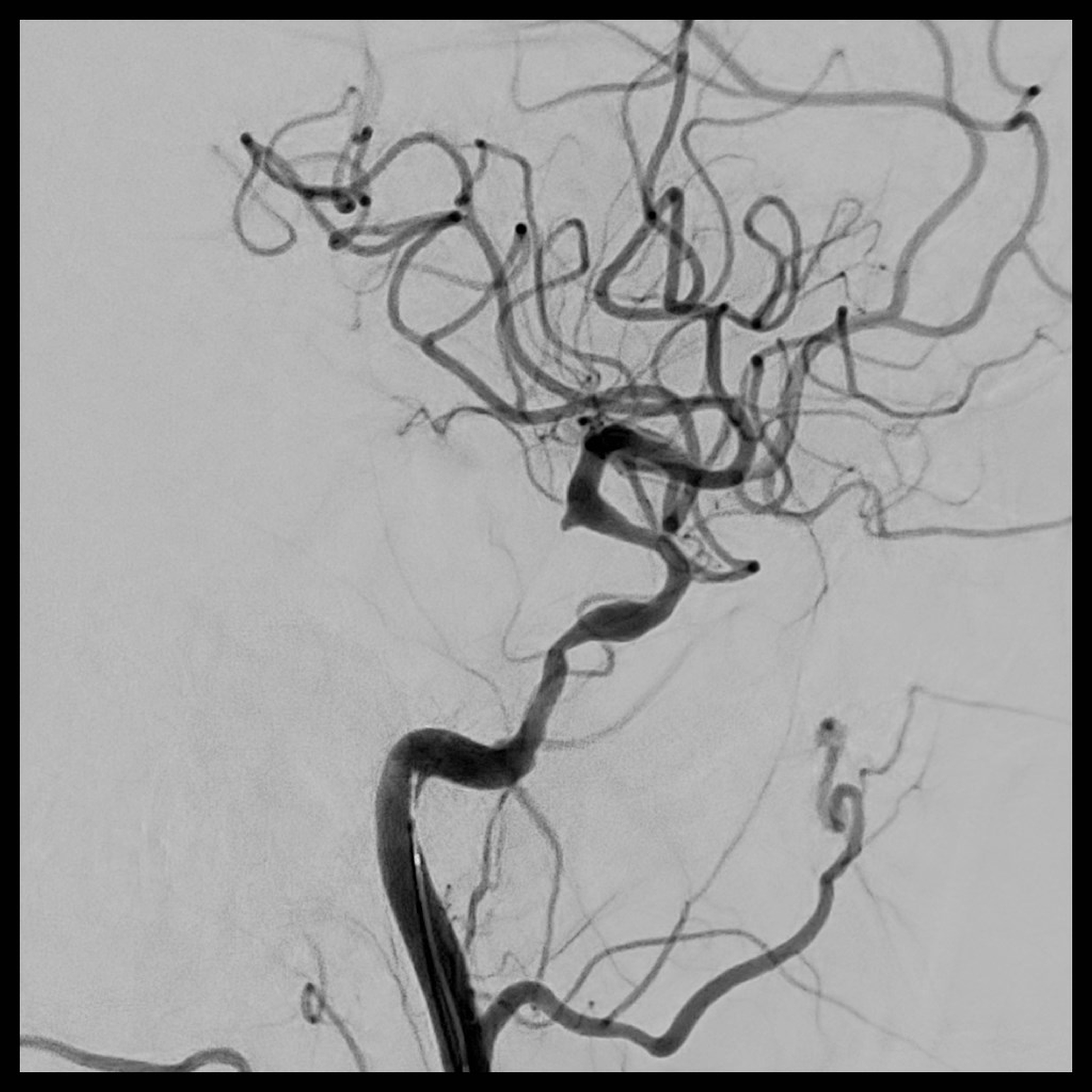

Supplement: PRESENTATION 1 [file Presentation_1.ZIP › 4b_Patient4_postPTA.jpg]
